# The ReSiT study (reducing sitting time): rationale and protocol for an exploratory pilot study of an intervention to reduce sitting time among office workers

**DOI:** 10.1186/s40814-017-0191-2

**Published:** 2017-11-28

**Authors:** Benjamin Gardner, Stephen Dewitt, Lee Smith, John P. Buckley, Stuart J. H. Biddle, Louise Mansfield

**Affiliations:** 1 Department of Psychology, Institute of Psychiatry, Psychology and Neuroscience, King’s College London, Room 2.11, Addison House, Guy’s Campus, London, SE1 1UL UK; 2 The Cambridge Centre for Sport and Exercise Sciences, Department of Life Sciences, Anglia Ruskin University, Cambridge, UK; 3 Institute of Medicine, University of Chester, Chester, UK; 4 University of Southern Queensland, Queensland, Australia; 5 Department of Life Sciences, College of Health and Life Sciences, Brunel University, Uxbridge, UK

**Keywords:** Sedentary behaviour, Sitting, Physical activity, Behaviour change, Sit-stand desks

## Abstract

**Background:**

Desk-based workers engage in long periods of uninterrupted sitting time, which has been associated with morbidity and premature mortality. Previous workplace intervention trials have demonstrated the potential of providing sit-stand workstations, and of administering motivational behaviour change techniques, for reducing sitting time. Yet, few studies have combined these approaches or explored the acceptability of discrete sitting-reduction behaviour change strategies. This paper describes the rationale for a sitting-reduction intervention that combines sit-stand workstations with motivational techniques, and procedures for a pilot study to explore the acceptability of core intervention components among university office workers.

**Methods:**

The intervention is based on a theory and evidence-based analysis of why office workers sit, and how best to reduce sitting time. It seeks to enhance motivation and capability, as well as identify opportunities, required to reduce sitting time. Thirty office workers will participate in the pilot study. They will complete an initial awareness-raising monitoring and feedback task and subsequently receive a sit-stand workstation for a 12-week period. They will also select from a ‘menu’ of behaviour change techniques tailored to self-declared barriers to sitting reduction, effectively co-producing and personally tailoring their intervention. Interviews at 1, 6, and 12 weeks post-intervention will explore intervention acceptability.

**Discussion:**

To our knowledge, this will be the first study to explore direct feedback from office workers on the acceptability of discrete tailored sitting-reduction intervention components that they have received. Participants’ choice of and reflections on intervention techniques will aid identification of strategies suitable for inclusion in the next iteration of the intervention, which will be delivered in a self-administered format to minimise resource burden.

**Trial registration:**

ISRCTN29395780 (registered 21 November 2016)

**Electronic supplementary material:**

The online version of this article (10.1186/s40814-017-0191-2) contains supplementary material, which is available to authorized users.

## Background

Prolonged sedentary behaviour—i.e., any waking behaviour characterised by an energy expenditure of 1.5 metabolic equivalents or less, undertaken while sitting or lying down [1]—is associated with morbidity and premature mortality [2–4]. Sitting time has, for example, been linked to increased risk of diabetes, heart disease, and some cancers [5–8]. There is some evidence to suggest that the mortality risk associated with sitting may be mitigated by taking 60+ minutes of at-least-moderate daily physical activity [9]. However, this is likely to be an unrealistic public health target, because many people are both highly sedentary and physically inactive. Objective data from a nationally representative survey indicated that 95% of adults in England do not meet physical activity recommendations [10]. Of particular concern are desk-based office workers. The typical office worker is estimated to sit for around 6 h (6 h) per 8 h working day [11] and 10.6 h in total across a 16 h waking day [12]. With half of the UK workforce based in offices [13], workplace sedentary behaviour represents a major public health concern [14].

A recent expert consensus guideline recommended that office workers aim to stand for 2–4 h per 8 h working day [14]. Various behaviour change interventions have been developed to support office workers to reduce their sitting time [15]. Many of these have used motivational techniques; that is, methods that seek to enhance desk-based workers’ motivation to reduce their sitting time, or enable them to act on their motivation. For example, one intervention featured regular emails offering tips on sitting less, social media promotion of sitting reduction, workplace champions, post-of-decision prompts and management support [16]. Observed reductions in sitting time (of up to 30 min per working day [16, 17]) in trials of such interventions testify to the potential value of individual-level motivational strategies [17, 18].

One trial evaluated software that deactivated workers’ computers every 45 min, to facilitate breaks from desk-based computer work to engage in light physical activity [19]. Interviews suggested this approach yielded some benefits, including increased awareness of unhealthy sitting practices, but some participants experienced frustration at forced interruptions to their workflow. This suggests that the intervention, while potentially efficacious, may not have been acceptable to desk-based workers. Interventions that lack acceptability—i.e., intended recipients are unwilling to engage with it—are unlikely to be implementable. While one study documented some public resistance to the notion of reducing workplace sitting time [20], interview studies indicate that office workers would be willing, in principle, to reduce sitting time at work [16, 21]. However, for many, the primary motivation during working hours is to complete work tasks [21, 22]. Taking regular standing breaks can be unwelcome, because it reduces valuable working time [22].

Height-adjustable sit-stand workstations (SSWs) are generally acceptable to office workers, as they permit desk-based work in a standing or seated position, so minimising workflow disruption [16, 21, 23, 24]. Managers often express concern about the cost of SSWs [22], which at present cost ≥ £279 (US $375) per unit [25, 26]. Growing evidence of their efficacy for reducing sitting time [27], and associated benefits to workers’ health and wellbeing [28], may help to increase acceptability among managers in the long-term.

It may be fruitful to combine SSWs with motivational behaviour change strategies. This would ensure that workers have the environmental support necessary to undertake desk work when standing and the motivation and capability to displace sitting with standing at the desk and in other office settings. To our knowledge, only two interventions have adopted this approach, and both were associated with reductions in sitting time [29–32]. For example, in an Australian pilot trial, workers received SSWs and, in one-to-one personally tailored behavioural counselling sessions and phone call follow-ups with health coaches, techniques designed to support the translation of sitting-reduction motivation into action (e.g. goal setting, self-monitoring, problem solving [30–32]). The acceptability of components of this intervention was later explored in consultation with office managers and employees, to ensure that the intervention was implementable [33].

Adopting a participatory approach to intervention development and evaluation can be of benefit in developing effective interventions [34, 35]. Participant involvement in intervention design and implementation can help to highlight the individual, organisational and cultural contexts into which workplace sitting reduction initiatives must be embedded if they are to be acceptable, feasible and effective.

## The present study

Previous work attests to the potential efficacy of combining SSWs and motivational behaviour change strategies. Yet, few studies have adopted this approach, and only one has explored intervention acceptability among desk-based workers [30]. This paper presents the rationale for a new theory- and evidence-based intervention that combines SSWs with motivational behaviour change techniques, and a protocol for a pilot study to explore the acceptability of discrete components of a prototype of the intervention. The intervention aims to reduce sitting time, and increase standing and light activity, among office workers. The work reported here represents the early stages of a broader intervention development project.

The pilot study uses a single-group, parallel mixed-methods design, and is designed to inform subsequent refinement of intervention content, ahead of its translation into a format suitable for real-world implementation and evaluation in a randomised controlled trial. The study has two specific objectives: primarily, to inform decisions about which components to consider removing, retaining, or refining in a later iteration of the intervention, and secondarily, to inform statistical power analysis for a future trial of the intervention. The primary objective is served by qualitative interview data, which will explore the behaviour change strategies deemed by office workers to be acceptable and useful, and expectations and experiences of the intervention more broadly. We do not employ pre-specified quantifiable criteria for acceptability, but rather use qualitative analyses to explore responses relevant to acceptability from participants’ in-depth reflections on their experiences. The secondary objective is served by quantitative accelerometry data, which will document minutes spent sitting, standing and moving in the workplace before and after receiving the intervention.

The study is registered (ISRCTN29395780), and all procedures detailed in the protocol below have received approval from the King’s College London Psychiatry, Nursing and Midwifery Ethics Panel (LRS-16/17-3718).

## Methods

### Intervention development process

Our intervention is being developed in line with UK Medical Research Council guidance [36], with iterative stages of development, feasibility, piloting and evaluation. We have organised this work into six stages. Stage 1 (*problem analysis*) involves drawing on relevant theory and empirical literature to articulate a set of core assumptions as to why office workers sit for long periods and how they might be supported to reduce sitting. Stage 2 (*identification of content for an intervention prototype*) draws on outputs from stage 1, and previous literature reviews, to inform the selection of core behaviour change techniques, and the development of a rudimentary intervention prototype. The acceptability of the content of the prototype will be explored at stage 3 (*piloting the intervention prototype*). At stage 4 (*development of a scalable iteration of the intervention*), intervention content will be refined in light of insights from stage 3, and translated into a delivery format deemed realistic and feasible for large-scale implementation in office-based organisations. Stages 5 (*piloting the intervention*) and 6 (*implementing and evaluating the intervention*) will focus on effectiveness and implementation.

This paper reports the rationale behind the intervention (i.e. stages 1 and 2) and a protocol for an exploratory pilot study of the intervention prototype (stage 3). Stages 4–6, which are not described here, will be undertaken following completion of the pilot study.

### Problem analysis: why do office workers sit for long periods, and how might sitting time be reduced?

#### Theoretical framework

We framed our problem analysis using the capability, opportunity, motivation–behaviour (COM-B) model [37, 38], which was developed to portray the fundamental determinants of all human behaviour, and so transcends and can incorporate all theoretical perspectives. COM-B proposes that all behaviour requires capability, opportunity and motivation. These components may be deconstructed further into physical or psychological capability, social or physical opportunity and reflective or automatic (i.e. conscious or non-conscious) motivation. Disrupting sitting thus requires reducing workers’ capability, opportunity, or motivation for sitting, or enhancing these components as they relate to alternative actions (i.e. standing or moving).

#### Literature review

Drawing on previous research into understanding, predicting and reducing workplace sedentary behaviour [11, 12, 16–24, 28–30, 39], we formed three key assumptions regarding workers’ capability, opportunity and motivation to reduce sitting.

#### Assumption 1: workplace sitting is a non-conscious behaviour

Workplace sitting is, for most workers, not consciously motivated, but rather a predominantly non-conscious behaviour incurred by work tasks and characteristics of the office environment [21, 22, 40]. For example, workers report getting ‘distracted’ and ‘caught up’ in their work, which prevents them from taking breaks from sitting [22]. The non-conscious nature of sitting likely limits awareness of true sitting time, for two reasons. First, sitting may be a habitual response, to which workers pay little attention [41]. Habit theory proposes that repetition of an action (e.g. sitting) in a consistent context (e.g. when entering the office) leads, through associative learning of an action-context association, to the action being initiated automatically upon subsequent exposure to the context [42–44]. The habitual action can occur with little or no conscious awareness or motivation [45]. The stable, unchanging nature of the office environment is highly conducive to habit formation [46]. Second, people cognitively organise action such that sitting is unlikely to be a salient activity. All behaviours can be deconstructed into multiple sub-behaviours; any one work task (e.g. ‘writing a report’) can be broken down into smaller tasks (e.g. ‘sitting down at my desk’, ‘turning on my computer’, ‘opening my word processor’, ‘typing words’ [47]). Action Identification Theory hypothesises that people mentally represent actions at higher levels of abstraction, according to their purposes or consequences (e.g. ‘writing a report’), rather than attending to lower-level procedural intricacies (‘sitting down at my desk’ [48]). Workers are unlikely to consciously attend to the low-level actions (e.g. sitting, turning on the computer) that comprise more personally meaningful work activities (e.g. ‘having a meeting’, ‘checking emails’, ‘writing reports’), and so are less likely to recall these sub-actions. Indeed, evidence consistently shows that people struggle to accurately recall sitting time directly [49], but offer more reliable estimates when sitting is operationalised as time spent in more meaningful, typically seated activities [50, 51]. Disrupting sitting habits may depend on raising office workers’ awareness of their sitting patterns, and the contextual cues that prompt prolonged sitting [52, 53], to increase their motivation to tackle prolonged sitting.

#### Assumption 2: reducing sitting is of low priority to office workers

Workers have multiple, potentially competing goals at work. Although sitting reduction may be potentially valued, most workers are likely to prioritise completion of work tasks over reducing sitting time [22]. In the absence of infrastructure that facilitates performance of work tasks while standing or moving, workers are unlikely to reduce their sitting. SSWs provide a vital opportunity for displacing sitting with standing, with no adverse impact on work task completion [54].

#### Assumption 3: reducing sitting is of low priority to managers

Managers are likely to prioritise productivity, the achievement of organisational objectives and cost-effectiveness, over workers’ sitting [22, 55]. Interventions that impose considerable financial or time burden on managers, or workers, are unlikely to be widely implemented. Managers’ priorities can also impact on workers’ sitting reduction efforts, because effective workplace interventions depend on managerial support [30, 33]. Implementable workplace sitting reduction interventions must acknowledge, and be embedded within, the priorities, practices and culture of the modern office [55, 56].

Our first two assumptions informed the selection of behaviour change techniques for inclusion in our intervention prototype (stage 2), which will be administered to office workers in a face-to-face behavioural counselling session (stage 3). This resource-intensive delivery method is unlikely to fit with managers’ priorities, and so, at stage 4, our third assumption will be addressed, through the translation of intervention content into a format suitable for large-scale implementation.

### Identification of content for intervention prototype

The intervention comprises three core components: an initial monitoring and feedback phase, provision of tailored sitting-reduction techniques and fitting of a height-adjustable SSW. A more detailed account of these components is provided below (‘Intervention content’). In light of the non-conscious nature of workplace sitting (assumption 1), the intervention seeks to firstly raise office workers’ awareness of their true sitting time, and the tasks that incur prolonged periods of sitting [52]. Thus, workers will be asked to wear objective sitting monitors, and record their work activities, for a predefined period. By providing feedback on sitting and tasks commonly undertaken while sitting, we aim to enhance motivation to reduce sitting time, and potentially identify opportune moments for standing and movement. SSWs are provided as a means of ensuring participants have a readily accessible opportunity to displace sitting time with standing while completing desk-based tasks, so that sitting reduction does not conflict with work tasks (assumption 2).

Workers must recognise the value of reducing their sitting time and feel able to take the necessary steps to do so. Thus, the intervention adopts additional behaviour change techniques well-suited to changing perceptions of capability, awareness of opportunities, or motivation to reduce workplace sitting. Our recent review found that the most promising workplace sitting-reduction strategies included educating workers about the risks of sitting or benefits of standing, setting and reviewing behavioural goals, self-monitoring, problem-solving, and restructuring the social and physical environment [15]. Office workers will however likely differ in their motivation, and perceptions of capability and opportunity, and so these strategies must be tailored to individual needs. An employee with little motivation to sit less will, for example, likely require different intervention techniques to a motivated worker unable to identify opportunities. Thus, in a face-to-face behavioural counselling session, participants will be offered a ‘menu’ of behaviour change techniques, tailored to their self-declared capability, opportunity and motivation, from which they will select techniques that they expect will be most useful for reducing their sitting time.

### Piloting the intervention prototype: a study protocol

#### Study design

The study uses an exploratory, single-group parallel mixed-methods design, to investigate the acceptability of components of our workplace sitting-reduction intervention prototype, among a sample of office workers at a London university. Universities possess a range of office environments for desk-based occupations, with workers from across the socioeconomic spectrum. The experiences of university workers are therefore likely to be relevant to desk-based workers in a range of other employment settings.

We will assess participants’ experiences of the intervention over time via semi-structured interviews, conducted at three points (1, 6 and 12 weeks post intervention) over a 12-week period. Additionally, all participants will be fitted with an accelerometer-inclinometer which distinguishes sitting and standing time, prior to and following the intervention, and a week prior to the 6- and 12-week follow-ups. This will allow quantification of changes in sitting, standing and activity following the intervention.

#### Participants

**Fig. 1:**
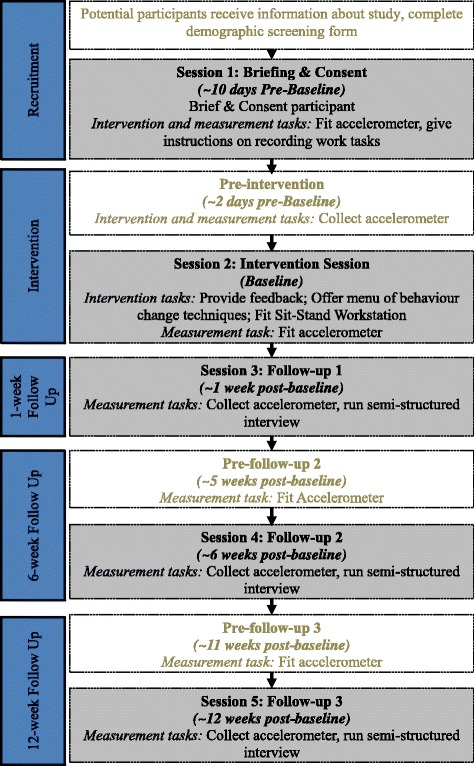
Participant flow

Desk-based workers (*N* = 30) will be recruited. Our sample size is pragmatic; a sample size of 30 is conventionally deemed adequate for single-group pilot studies, as it permits collection of enough useful data while minimising research costs [57]. It is also appropriate in qualitative research of this kind, for recruitment of a sample with a broad demographic profile [58] (see Fig. 1).

#### Eligibility criteria

Participants will be office- and desk-based workers aged 18 years or over who self-declare working at least 3 days per week, and whose job requires them to sit at a workstation of which they are the sole user for the majority of their typical working day. This will ensure they have sole access to the SSW for a sufficient period of time during the intervention to implement the behavioural strategies.

Workers with a physical condition which prevents standing for prolonged periods (e.g. musculoskeletal, pain, pregnancy) will be excluded. Participants must not have taken part in similar workplace standing research previously, nor ever used a SSW for two or more consecutive days. They also must not intend to leave the employer of the host site or to take an absence for longer than 10 consecutive work days for the duration of the study.

#### Recruitment procedure

The study will be advertised via posters at the host site and fortnightly all-staff circular emails. Participants, who self-report meeting eligibility criteria, as stated in study advertisements, will be emailed an information form and demographic questionnaire. The demographic questionnaire will allow us to not only record the demographic profile of our sample but also, if more than 30 eligible potential participants express interest, to screen and select participants to ensure a diversity of age and occupational seniority. They will have the opportunity to discuss the project by phone with a researcher prior to deciding whether to take part.

Due to limited staff resources, recruitment will be staggered such that no more than one participant enters the study on any one day. Participant screening would thus be responsive; the research team would compare the demographics of those expressing interest against those of participants already enrolled into the study. Otherwise, eligible participants from demographic categories judged by the research team to be potentially over-represented in the sample—for example, early-career post-doctoral academic research workers—will be placed on a reserve list, and only consented if fewer than 30 participants can be identified.

#### Study procedure

Unless otherwise stated, all sessions described below will take place in a private meeting room local and convenient to the participant, in the workplace (e.g. their office, or a local meeting room). One researcher—a post-doctoral psychologist—will run all sessions.

##### Session 1 (10 days pre-baseline)

Potential participants who complete the questionnaire will be invited to a face-to-face meeting at which they will be informed of the study timeline and procedures, and invited to complete a consent form. Those who consent will be fitted with an activPAL accelerometer/inclinometer device (PAL Technologies, Glasgow, UK), wrapped in a nitrile dressing and covered using a waterproof medical grade dressing, for continuous wear for 7 days. The researcher will instruct and demonstrate to the participant how to fit the device to the centre of the lower right thigh.

Participants will be asked to monitor the work tasks they undertake for the following week, using task categories agreed between the researcher and participant (e.g. ‘emailing’, ‘phone calls’, ‘word processing’, ‘scanning’, ‘printing’). During this one-week period, participants will be sent two emails daily, once in the middle of the working day and once around the end of the working day, inviting them to log their tasks by replying to the email. The email will require information on which of the agreed task categories have been undertaken during each hour of the working day up to that point. At the end of the final day of monitoring (the fifth work day), participants will receive an additional email requesting an estimation of the total time they have spent sitting during the previous five days of monitoring from 9 am to 5 pm including their lunch break. Eight days later (allowing an extra day for the accelerometer to complete data collection), the researcher will collect the accelerometer.

##### Session 2: intervention (baseline)

Ten days after session 1 (allowing 2 days for synthesis of accelerometry and work-task data), a second session will be held at which the motivational component of the intervention and SSW will be administered. Participants will be provided with feedback on their sitting time and their tasks over the preceding week. They will be asked which of the following three statements, derived from the COM-B questionnaire [37], is most applicable to them: “I do not feel capable of reducing my sitting at work” (capability); “I do not feel I have the opportunity to reduce my sitting at work” (opportunity); “I do not feel motivated to reduce my sitting at work” (motivation). They will then be offered a selection of behaviour change techniques, tailored to their responses to the questions. This portion of the intervention session will be conducted in a private meeting room, and while participants will be free to ask questions at any point, we expect it to take a maximum of 40 min. Participants will also fit themselves with the accelerometer, for data collection purposes only, for a further 7-day wear period.

Following this, a height-adjustable desk-mounted SSW unit will be fitted to their office desk, and they will be provided with ergonomic instructions and accompanying tips. This portion will take a maximum of 20 min.

##### Sessions 3–5: follow ups 1–3 (1, 6, 12 weeks post-baseline)

Participants will be visited on three further occasions (as close to 1, 6 and 12 weeks post-baseline as possible). At each visit, the researcher will collect the accelerometer (fitted 1 week prior by the researcher) and conduct a semi-structured interview. At the final session (12 weeks post-baseline), the SSW will be removed.

#### Intervention content

Our intervention prototype comprises three, sequentially administered components: (a) an initial phase of monitoring and feedback on existing sitting patterns; (b) a ‘menu’ of behaviour change techniques from which participants select techniques tailored to their needs; and (c) provision of a height-adjustable desk for a 12-week period. Intervention content is described more fully, using terminology from the BCT Taxonomy v1 [59], in Additional file 1.

##### Monitoring and feedback

The researcher will provide verbal and visual feedback on participants’ objectively recorded average daily sitting time over the monitoring week (i.e. between sessions 1 and 2), with comparison to their subjective estimate of their sitting time. They will be shown a bar graph, created in Microsoft Excel, of raw data extracted from the accelerometer depicting the average proportion of each hour of the working day (0900 am–1700 pm) spent sitting, standing and walking. This will identify and stimulate discussion of prolonged periods of sitting. The researcher will discuss the task record for the previous week, as well as activity levels during their commute, lunch time and any breaks throughout their working day. The researcher will also provide personalised feedback on the tasks and times of day apparently most conducive to sitting, standing or light activity. Participants will be told of recent research that has linked prolonged periods of sitting and increased risks of heart disease and diabetes [5–8], and will be provided with a set of expert-consensus guidelines for reducing sitting [14]. The aim of this component is to raise awareness of true sitting time and its health implications, and highlight personally relevant work tasks that incur sitting versus those associated with more movement.

##### Menu of behaviour change techniques

Next, participants will receive access to a set of potential behaviour change techniques tailored to their self-declared COM-B barriers to sitting-reduction. Responses to COM-B questions will determine which techniques will be focused upon. After selecting barrier-matched techniques, participants will be offered the option of viewing non-barrier-matched techniques.

##### Sit-stand workstation

Participants will receive a VariDesk Pro Plus 30 desk-mounted unit (Varidesk, TX, USA; £325 [US$405]) for the 12-week intervention period. The VariDesk unit has been selected because it is height-adjustable and noiseless, allowing both sitting and standing work with minimal disruption to others. Participants will be instructed in appropriate, safe use of the workstation by the researcher, who has received Display Screen Equipment training. A poster with images displaying ergonomically optimal desk use will be placed near the workstation, to be visible when standing or sitting. Participants will also be offered a range of tips regarding the use of the desk, including tips on physical comfort and environmental strategies to facilitate sitting reduction (e.g. leaving the desk in the raised position at the end of the workday). If it is not possible to fit the workstation during session 2, it will be fitted at a subsequent appointment as soon as possible after session 2.

A summary of key points of the session will be emailed to participants the following day. They will also be offered regular emails containing key points from the session, to serve as motivational boosters and reminders of the session, for the 12-week intervention period. Participants will be asked, in session 2, whether there are specific points that they wish to have reiterated in these emails, and how frequently they wish to receive them.

#### Qualitative data and analysis

##### Intervention session (session 2) and semi-structured interviews (sessions 3–5)

The intervention session, and semi-structured interviews undertaken at 1, 6 and 12 weeks post-baseline, will all be digitally recorded and transcribed verbatim for analysis. While participants will not be interviewed in the intervention session, their utterances will be treated as study data as they may reveal expectations of the intervention, and decisions underpinning intervention technique choices.

The three semi-structured interviews will cover the following: participants’ experiences of sitting and standing since the previous meeting; the usefulness of the sitting-reduction techniques delivered to them, including views towards the SSW; their use of the SSW and adherence to the techniques, and any suggested improvements or amendments; their perceptions of their capability, motivation and opportunity to reduce their sitting; and the conduciveness of the physical and social office environment to sitting-reduction (see Additional file 2). At 1-week post-baseline only, the interview will also cover motives for and expectations of participation and reducing sitting.

##### Analysis

Qualitative data will be analysed using inductive thematic analysis procedures, from a realist epistemological perspective [60]. Analysis will generate a set of themes that reveal which intervention components appeared acceptable and why, and which require refinement or removal from the intervention. Data will also reveal participants’ underlying beliefs, attitudes and values regarding sitting, standing and moving in the workplace, which likely act as barriers to or facilitators of workplace sitting reduction [61].

#### Quantitative data and analysis

##### Demographics

Gender, age, postcode, ethnicity, highest qualification, annual income, presence of disability and job title data will be self-reported for sample description purposes.

##### Sitting, standing and activity

Sitting, standing and sit-stand transition data will be recorded using the activPAL micro accelerometer-inclinometer (PAL Technologies Ltd., Glasgow, UK), for 7-day wear periods. The activPAL micro is small (53 × 35 × 7 mm), lightweight and provides accurate measures of sitting time and sit-to-stand transitions per hour, as validated against direct observation in free-living environments [62–64]. Sitting time data will be used to both analyse the success of the approach and to provide feedback on sitting times to participants during the intervention session.

##### Analysis

Quantitative data (accelerometry) will be extracted using specialist software designed for use with the activPAL (activPAL™ Professional v7.2.32; PAL Technologies Ltd., Glasgow, UK). Daily data will be entered into analysis only where devices have been worn for 24 h (0000 to 2359). All data will be visually inspected to identify any unusual episodes (e.g. few steps recorded, indicating the device may have malfunctioned or not have been worn), and days containing such episodes will be excluded from analyses.

Time-stamped data will be summarised in 15 s intervals and analysed in hourly intervals. Accelerometry outcomes of interest, which include daily times spent sitting, standing and stepping, frequency of sit-stand transitions and step counts, will be calculated for participants with available data on at least three weekdays and at least one weekend day. ActivPAL data distributions from a previous large observational study suggest office workers are most likely to be awake between 0700 and 2300 [12]. Daily sitting will thus be categorised as sedentary time accumulated between 0700 and 2300. Data from each 7-day observation period will be summarised for each participant, and aggregated across all participants, using descriptive statistics (means, confidence intervals). Changes in group-level aggregated accelerometry outcomes over time will be assessed using mixed-effects ANOVA, to allow for multiple measures from each participant at each of four timepoints.

## Discussion

Evidence increasingly suggests that sitting time is a risk factor for morbidity and early death [2–4]. We are developing a novel sedentary behaviour reduction intervention, based on a theory- and evidence-based analysis of why office workers sit and how best to reduce sitting. The intervention combines two approaches—provision of SSWs, and motivational support—that have separately shown promise for reducing sitting time [15, 27]. We will pilot a prototype of the intervention among a sample of 30 office workers at a UK university. Novel elements of our intervention are that the motivational support intervention component is tailored to theory-derived determinants of sitting reduction, and that, in engaging participants in selecting from a menu of intervention techniques, our participants will co-design their intervention and its implementation. This participatory approach may enhance intervention effectiveness [34, 35]. Our pilot study will be the first, to our knowledge, to explore direct feedback on the acceptability of discrete tailored sitting-reduction intervention components. Acceptability is an important determinant of intervention success [37], but has been largely overlooked in the development and testing of sitting-reduction interventions to date. Results will inform the refinement of intervention content, as a subsequent stage in an overarching intervention development project.

Applying theory to intervention design allows scientific knowledge about behaviour change to be used in specifying the techniques most likely to change behaviour, and the mechanisms through which such change might be achieved [65]. Yet, few workplace sitting-reduction interventions evaluated to date have been explicitly theory-based [15]. There are multiple ways in which theory can inform interventions [66]. Drawing on the COM-B model [38], our intervention delivers change techniques tailored to participants’ self-reported capability, opportunity and motivation to reduce sitting. There are limitations to this approach; participants may not be aware of the true barriers to their sitting [67], and so the techniques they choose to receive may lack efficacy for reducing their sitting time. Nonetheless, it seems prudent to identify the intervention content with which office workers are most (or least) likely to engage. Interventions that are not acceptable to office workers are unlikely to be implementable.

We will adopt an exploratory qualitative approach to investigate intervention acceptability, probing participants’ reflections to identify elements they did or did not find useful, interesting, engaging, or otherwise pertinent. We did not consult directly with office workers to generate intervention content at the outset of the intervention development process. Studies reporting workers’ ideas for sitting reduction have tended to yield similar suggestions (e.g. SSWs, computer prompts, standing meetings, removing chairs, education [16, 21, 22, 39]). We have incorporated many of these suggestions into our intervention as advice for identifying and seizing opportunities to sit less. We will instead explore the acceptability of core components of our intervention among workers who have attempted to engage with it. One limitation is that our qualitative methods will not quantify acceptability, precluding evaluation against a predetermined threshold. Conversely, however, qualitative methods allow for in-depth coverage of the reasons underlying acceptability [61]. This will help us to identify whether and how discrete components require modification in the next iteration of the intervention. Furthermore, views expressed by our intervention recipients towards a sitting-reduction intervention have the potential to generate new knowledge of office workers’ underlying beliefs, attitudes and values towards workplace sitting and activity, which may not be revealed by direct questioning [61].

One potential limitation of our study is that acceptability is explored among intervention recipients only. The feasibility of workplace sitting-reduction interventions depends on acceptability not only among office workers but also managers [30, 33]. Senior management at the study site—a UK university—may have a more positive and open attitude towards testing novel, evidence-based sitting-reduction interventions than other employers. Research has documented doubts among employers about the benefits of sitting reduction [22]. Furthermore, the intervention prototype we will test is resource-intensive, requiring one-to-one behavioural counselling from a trained psychologist. This is unlikely to be scalable; office managers may not be willing to fund provision of such support or may not want workers to attend such sessions during working hours. Indeed, office workers may not be willing to attend lengthy one-to-one appointments if they are seen to reduce time available for pressing work tasks [22]. However, the present study represents a step towards developing a scalable intervention acceptable to employers and workers alike. We will use the findings from the present study to inform a refinement of the intervention that can be delivered in a less resource-intensive way. Tailored self-administered computer-based interventions, for example, can effectively mimic face-to-face behavioural counselling [68]. While we note that a recent computer-based sitting-reduction intervention was found to have no impact on objectively measured sitting time [24], this may have been due to the selection of content included in the intervention, rather than the delivery format. At a later stage in the development process, we intend to translate content of the intervention prototype that is deemed acceptable in the present study into a self-administered format. As a precursor to further intervention development work, an additional limitation of the present study is that participants’ views will necessarily be based on experiences of one-to-one delivery of intervention components. While we will attempt to identify insights that would likely apply across delivery methods, we may need to conduct further work to determine whether amending the delivery format affects the acceptability of intervention components.

Another limitation of our study is that it targets sitting reduction primarily by addressing the needs of individual workers. Such an individual-level conceptualisation of sitting reduction could be argued to neglect broader determinants of workplace sitting behaviour. Ecological models portray sedentary behaviour as the product of a complex interplay of individual, organisational and environmental factors [69]. Achieving sustainable reductions in workplace sedentary behaviour may require not only changes in workers’ motivation and capability, and the provision of more opportunities, but also commitment from senior management to the creation of organisational and cultural norms supporting standing and light movement [33]. The aims of the present study are, however, consistent with a broader ecological approach. Managers are unlikely to want to commit resources to support intervention strategies that are shown to lack acceptability among office workers [22]. Our study will help to identify discrete sitting-reduction intervention components with which office workers are most willing to engage.

### Trial status

The trial is currently in the recruitment and data collection phase.

## Additional files

Additional file 1: Table S1. Intervention content provided in session 2: description and component behaviour change techniques. (DOC 35 kb)

Additional file 2: Appendix 1. Interview schedules. (DOCX 25 kb)

## Abbreviations

ANOVA: Analysis of variance
BCT: Behaviour change technique
COM-B: Capability, opportunity, motivation-behaviour model
ISRCTN: International Standard RCT Number Register
RCT: Randomised controlled trial
SSW: Sit-stand workstation
