# Supplementary material for: The ReSiT study (reducing sitting time): rationale and protocol for an exploratory pilot study of an intervention to reduce sitting time among office workers
Source: Pilot Feasibility Stud. 2017 Nov 28;3:47. doi: 10.1186/s40814-017-0191-2 (PMC5704376; doi:10.1186/s40814-017-0191-2)
Supplement: Supplementary file 1 — Intervention content provided in session 2: description and component behaviour change techniques. (DOC 35 kb) [file 40814_2017_191_MOESM1_ESM.doc]

**Additional File 1. Table S1.** Intervention content provided in Session 2: description and component behaviour change techniques

| *Intervention phase* | *Content* | *Behaviour change techniques* |
| --- | --- | --- |
| Monitoring and feedback | *(Pre-Session 2:)* Participants wear accelerometer-inclinometer to track sitting, standing and movement time, and complete task diary  - Provision of a bar graph feeding back participant’s average daily sitting, standing and walking time  - Feedback on tasks or times of day associated with prolonged sitting, or movement  - Researcher-provided description of research linking sitting to health risks | - Self-monitoring of behaviour  - Feedback on behaviour  - Information about antecedents  - Information about health consequences |

| Menu of Behaviour Change Techniques* | *Opportunity-linked techniques**  - Tips for incorporating more standing in to the work day (e.g. speak face-to-face rather than email, stand in meetings, stand while using phone, take walking lunch breaks, use the stairs)  *Capability-linked techniques**  - Advice on setting specific and achievable behavioural and outcome goals for sitting, standing or activity time  - Advice on identifying and planning for contexts conducive to sitting less  - Information on psychology of how actions such as sitting become automatic responses to specific contexts through learned associations, and importance of forming associations between standing and moving and office cues  - Advice on how to shield sitting-reduction intentions from derailment in specific contexts  - Information on psychology of overcoming unwanted automatic responses to situational cues  *Motivation-linked techniques**  - Further summary of research evidence on links between sitting less, standing more and health benefits  - Testimonies from other successful standing workers, including references to famous standing workers (e.g. Hemingway, Churchill), and quotes from participants in previous qualitative studies  - Summary of apparent misconceptions about sitting, standing and moving (e.g. sitting risk is solely attributable to physical inactivity), and evidence-based rebuttals of these  *All participants:*  - Personalised email or SMS reminders (for delivery after Session 2), featuring information received by participant during Session 2 | - Instruction on how to perform the behaviour  - Goal setting (behaviour)  - Goal setting (outcome)  - Action planning  - Habit formation  - Problem solving  - Habit reversal  - Information about health consequences  - Information about others’ approval  - Information about health consequences  - Prompts/cues |
| --- | --- | --- |
| Provision of sit-stand workstation | - Provision of workstation  - Instructions for ergonomic use  - Tips for encouraging desk use (e.g. leave in standing position at end of day) | - Restructuring the physical environment  - Adding objects to the environment  - Instruction on how to perform the behaviour |

* These clusters of techniques are initially presented according to whether participants self-report opportunity, capability or motivation to be their primary barrier to sitting reduction. After receiving the barrier-linked techniques, all participants are given the option of receiving techniques linked to other barriers.
