# Supplementary material for: The ReSiT study (reducing sitting time): rationale and protocol for an exploratory pilot study of an intervention to reduce sitting time among office workers
Source: Pilot Feasibility Stud. 2017 Nov 28;3:47. doi: 10.1186/s40814-017-0191-2 (PMC5704376; doi:10.1186/s40814-017-0191-2)
Supplement: Supplementary file 2 — Interview schedules. (DOCX 25 kb) [file 40814_2017_191_MOESM2_ESM.docx]

**Additional File 2. Appendix 1. Interview schedules**

**Interview 1 (Session 3)**

1. Motivation to participate
   1. Why did you want to take part in this study?
2. Motivation to stand
   1. Before we began this study, did you want to stand or were you sceptical about standing at work?
   2. Why were you interested in standing at work initially?
   3. What do you now understand the benefits of standing at work to be?
3. Expectations about standing
   1. What were your expectations about standing at work before our previous session?
   2. Have your expectations about standing at work changed since our previous session?
4. Experience of standing
   1. Describe your standing experience over the previous week since our last session
   2. When / in what situations did you stand at work over the last week?
   3. Did you discover any other methods or tools for reducing your sitting time at work over the previous week(s)?
5. Capability / Opportunity to stand
   1. Did anything restrict you from standing over the previous week(s)?
   2. Did anything facilitate your standing?
   3. Would anything make it easier for you to stand, or make you more willing to stand?
   4. Have you found any particular tasks more conducive to sitting versus standing
   5. Have you found any particular times of days more conducive to sitting versus standing?
6. Workplace context for standing, norms and habits in meetings
   1. Did it seem ‘normal’ to stand at work or was it a new experience for you?
   2. How did others in your office (if applicable) react to you standing?
   3. Did you stand in any other aspects of your job?
   4. Do you think the workplace is an appropriate context to stand?
   5. Was your manager supportive of your standing at work?
7. Questions specific to each element of individual intervention chosen (e.g. goal setting, tips for standing)
   1. What was your experience / How helpful did you find X?
   2. Did you experience any difficulty in implementing X?
   3. Did you experience any success in implementing X?
   4. What could be improved about X?
   5. The aim of our study is to look at what seems realistic and useful for reducing sitting time in people who work at desks. Would you recommend X to other desk users who usually sit?
8. Additional Questions.
   1. Anything in the intervention particularly useful / memorable?
   2. Beneficial to see strategies again?

**Interviews 2 and 3 (Sessions 4 and 5)**

1. Motivation to stand
   1. What do you now understand the benefits of standing at work to be?
2. Expectations about standing
   1. Have your expectations about standing at work changed since our previous session?
3. Experience of standing
   1. Describe your standing experience over the previous week(s) since our last session
   2. When / in what situations did you stand at work over the last week(s)?
   3. Did you discover any other methods or tools for reducing your sitting time at work over the previous week(s)?
4. Capability / Opportunity to stand
   1. Did anything restrict you from standing over the previous week(s)?
   2. Did anything facilitate your standing?
   3. Would anything make it easier for you to stand, or make you more willing to stand?
   4. How did others in your office (if applicable) react to you standing?
   5. Did you stand in any other aspects of your job?
   6. Have you found any particular tasks more conducive to sitting versus standing
   7. Have you found any particular times of days more conducive to sitting versus standing?
5. Questions specific to each element of individual intervention chosen (e.g. goal setting, tips for standing)
   1. What was your experience / How helpful did you find X?
   2. Did you experience any difficulty in implementing X?
   3. Did you experience any success in implementing X?
   4. What could be improved about X?
   5. The aim of our study is to look at what seems realistic and useful for reducing sitting time in people who work at desks. Would you recommend X to other desk users who usually sit?
